# Human Infections Caused by Flavobacteriales: Clinical Characteristics and Antimicrobial Susceptibility Patterns

**DOI:** 10.3390/idr18040080

**Published:** 2026-07-30

**Authors:** Grant Whitmer, Maria Joyce

**Affiliations:** 1 Division of Infectious Diseases, Department of Medicine, Duke University School of Medicine, Durham, NC 27710, USA; maria.joyce@va.gov; 2 Department of Pathology, Duke University School of Medicine, Durham, NC 27710, USA; 3 Durham VA Health Care System, Durham, NC 27705, USA

**Keywords:** *Chryseobacterium*, *Elizabethkingia*, *Myroides*, *Capnocytophaga*, *Bergeyella*, carbapenem, aminoglycoside, resistance

## Abstract

Background/Objectives: Bacteria of the order Flavobacteriales are environmental Gram-negative bacilli. Several organisms within the order represent rare but clinically important causes of infection in human hosts, particularly in immunocompromised and hospitalized individuals. Eight genera have been implicated in human infections: *Elizabethkingia*, *Capnocytophaga*, *Chryseobacterium*, *Myroides*, *Bergeyella*, *Flavobacterium*, *Empedobacter*, and *Weeksella*. Despite their clinical relevance, there has been, as yet, no consolidated summary of clinical characteristics or resistance patterns across pathogens within the order. Methods: This narrative review examines the currently published data on human infections caused by organisms within Flavobacteriales. 63 sources, including 57 peer-reviewed articles, were included. Results: The clinical associations of infections caused by each of the above genera are discussed. In addition, available antimicrobial resistance data for the same organisms are summarized. Knowledge gaps are identified, including the lack of dedicated antimicrobial susceptibility breakpoints and limited data on rare categories of organisms. Conclusions: Human pathogens within Flavobacteriales display a number of commonalities, including a predilection for immunocompromised hosts, and frequently display resistance to carbapenems and aminoglycosides. Additional studies to better characterize these pathogens and optimize the approach to their treatment are strongly warranted.

## 1. Introduction

Flavobacteriales is a taxonomic order of bacteria consisting of environmental Gram-negative bacilli. Most are oxidase-positive and nonmotile, though some have been noted to display gliding motility [1,2]. Production of colored pigments in isolates is frequently seen [3], which is reflected in the order’s name: ‘Flavo’ is a Latin root word meaning ‘yellow’.

Multiple changes in taxonomic classification of bacteria within the order Flavobacteriales have occurred. While historically, a grouping system based on phenotypic characteristics was often utilized [4], the development of molecular identification methods has allowed for more precise distinctions amongst these organisms. Accordingly, there have been multiple recent shifts in genus and species classification within the order. For instance, the organism *Elizabethkingia meningoseptica* was classified as *Chryseobacterium meningosepticum* prior to its reclassification in 2005 [5]. Flavobacteriales as an independent order was initially described in 2011 [6] and currently contains at least fifteen separate families [7].

Within Flavobacteriales, multiple genera contain organisms known to cause infections in humans. These genera belong to the families Flavobacteriaceae (*Capnocytophaga*, *Flavobacterium*, and *Myroides*) and Weeksellaceae (*Elizabethkingia*, *Chryseobacterium*, *Bergeyella*, *Empedobacter*, and *Weeksella*) [7]. Despite the clinical importance of these organisms, the literature on infections caused by organisms within the order is largely limited to case reports and case series, without a consolidated summary of clinical characteristics or antimicrobial resistance patterns yet available. This narrative review examines the available published data on these pathogens, with the goal of identifying current knowledge gaps and generating questions for future research initiatives.

## 2. Methods

This study comprises a narrative and descriptive review of the available medical literature. The literature search included English-language peer-reviewed articles relevant to the microbiology, epidemiology, antimicrobial susceptibility patterns, resistance mechanisms, clinical characteristics, diagnosis, or treatment of infections caused by clinically relevant members of the order Flavobacteriales. Original research articles, observational studies, case series, case reports, and relevant review articles indexed on PubMed or Google Scholar were considered. Articles were identified in PubMed and Google Scholar using targeted keywords with appropriate Boolean operators; additional references were identified through manual review of citations from identified relevant articles, when appropriate. Additional sources identified outside of those indicated above include indexed abstracts, US State and Federal government webpages, published textbooks, and publicly available online material from relevant professional societies. No limitation on year of publishing was placed on included sources, though where feasible the most recent pertinent sources were included. Sources were excluded if not published in the English-language, if they focused only on environmental or veterinary isolates without relevance to disease in humans, or lacked clinically applicable data.

In the first draft of the manuscript, 60 sources were included: 54 peer-reviewed articles, a single indexed abstract, a single US Federal Government website, a single website from the Wisconsin Department of Public Health, a single published textbook, and two webpages published by relevant professional societies. Following the initial literature search, three additional peer-reviewed articles were identified through targeted searches in order to ensure comprehensive coverage of relevant topics in the discussion section.

Formal study quality assessment and quantitative synthesis were not performed for the purposes of this narrative review, and literature selection was based on relevance to the review objectives and the clinical applicability of the findings. Citations were organized and collated using the Mendeley Reference Manager (Version 2.143.0). No unpublished data were obtained, analyzed or otherwise included in this narrative review.

## 3. Results

### 3.1. Clinical Characteristics of Infections by Organisms Within the Order Flavobacteriales

Pathogens of the order Flavobacteriales seem to have a strong predisposition to affect immunocompromised and hospitalized patients. This pattern is seen clearly in the pathogens of the order *Elizabethkingia*. For instance, in a review of 11 cases of *Elizabethkingia anophelis* bacteremia in an American academic hospital, all patients had significant medical comorbidities as well as prior exposure to the healthcare system [8]. *Elizabethkingia meningoseptica* has been implicated in outbreaks of neonatal meningitis, as well as sepsis in older and immunocompromised patients [9,10,11,12,13].

Among a review of 71 reported cases of infection caused by *Chryseobacterium indologenes*, significant medical comorbidities were present in more than half of patients, and indwelling medical devices, including central venous catheters, were involved in 39% of cases. Bacteremia and pneumonia were the two most common sites of identified infection in this analysis [14]. In a review of case reports of *C. gleum* infection, blood, urine, and respiratory sources were found to predominate [15].

Organisms within the genus *Myroides* are similarly known to cause infections in immunocompromised patients [16]. In a retrospective cohort study of 21 hospitalized patients with *Myroides* sp. infections, skin and soft tissue infections, osteomyelitis, and urinary tract infections were the most frequent manifestations. Bacteremia was present in 28.5% of infections included in this analysis [17]. There are growing reports of serious *Myroides* infections in seemingly immunocompetent individuals as well [18,19].

Reported infections with *Empedobacter* spp. are smaller in number, but are noted to affect both immunocompetent and immunosuppressed individuals. There has been at least one report of neonatal meningitis caused by *E. brevis* [20], as well as multiple case reports of bacteremia from *E. brevis* and *E. falsenii* in immunocompromised individuals [21,22]. *E. brevis* has also been implicated in an outbreak of twelve cases of endophthalmitis after ophthalmologic surgery, eleven of which required subsequent vitreous surgery as part of treatment [23].

Only rare case reports of infection with *Weeksella virosa* are available in the literature; a review of sixteen of these infections noted that four patients carried diagnoses of end-stage renal disease, three had diabetes mellitus, and three had an active malignancy. Notably, 10 of the 13 cases in which the sex of the patient was reported occurred in women, and three occurred in the setting of urologic or gynecologic comorbidities or pathology [24], which is a notable finding in that the vaginal carriage of this organism has been firmly established [25].

Cases of human infection with pathogens in the genus *Flavobacterium* are quite rare. *Flavobacterium ceti* has been reported as a causative pathogen in bacteremia in a patient with cirrhotic liver disease [26] as well as meningitis and bacteremia in a patient with diabetes in the setting of recent neurosurgical intervention [27]. *Flavobacterium lindanitolerans* was reported as the causative pathogen in multiple cases of meningitis: one in an elderly woman with a history of prior splenectomy and diabetes mellitus [28], and two in reportedly immunocompetent patients with a history of prior sinus or skull base surgery [29,30]. An outbreak of sepsis from *Flavobacterium* in an Iranian neonatal intensive care unit has also been reported and was linked to contaminated distilled water, though a species identification was not published in this case, and the availability of additional details from this outbreak is limited [31].

*Bergeyella* and *Capnocytophaga* spp. are known components of the oral flora in animals, including dogs and cats, and correspondingly have been linked directly to animal bite wounds. For example, a 2024 narrative review identified 26 cases of infective endocarditis caused by *Capnocytophaga* spp., of which more than half had either a predisposing animal bite or other associated exposure to the oral secretions of animals. Asplenia and neutropenia have also been identified as risk factors for the development of life-threatening sepsis from *Capnocytophaga* spp., and particularly from *C. canimorsus* [32]. Similarly, multiple case reports of infections with pathogens in the genus *Bergeyella*, including *B. zoohelcum*, have been linked directly to animal bites [33,34,35,36]. It has also been proposed that *B. cardium,* which has been identified as the causative organism in multiple cases of endocarditis, may be a component of the human oral microbiome [37].

Infections caused by organisms within Flavobacteriales seem to be associated with frequent mortality and morbidity. Both mortality and rates of inappropriate antibiotic treatment were high in a scoping review of studies of infections caused by *Chryseobacterium* [38]. In a descriptive analysis of a clonal outbreak of pan-resistant *Myroides odoratimimus* in a Turkish ICU, 19 of 27 patients ultimately died; notably, all included patients were also noted to have indwelling urinary catheters [39]. During the 2016 outbreak of *Elizabethkingia anophelis* in the US Midwest, the Wisconsin Department of Public Health reported 18 deaths among 63 confirmed cases, and an additional death among four probable cases [40]. In a retrospective study of seven cases of *E. meningoseptica* meningitis in neonates in a tertiary care center in India, two patients ultimately died and three survivors subsequently developed hydrocephalus [11].

### 3.2. Antimicrobial Susceptibility Patterns

Interpretation of antimicrobial susceptibility data in these pathogens is difficult; the Clinical and Laboratory Standards Institute continues to publish breakpoints for “Other Non-Enterobacterales” [41] but no genus- or species-specific breakpoints for pathogens in the genera described above. The European Committee on Antimicrobial Susceptibility Testing similarly does not have published breakpoints for any species within Flavobacteriales, instead offering guidance for situations in which susceptibility breakpoints are not available [42]. It has also been previously suggested that the E-test, disc diffusion, and agar dilution methods for antibiotic susceptibility testing of certain organisms within the order Flavobacteriales demonstrate an unacceptably high rate of error when compared with broth microdilution [43]. As such, the available antimicrobial susceptibility data reviewed below should be interpreted within these constraints.

Several pathogenic genera within Flavobacteriales appear to be intrinsically resistant to aminoglycoside antibiotics. Near-ubiquitous resistance has been reported in *Elizabethkingia* spp. [12,44,45], and in *Myroides* spp. [16,46]; resistance has also been reported to be very frequent in *Capnocytophaga* spp. [32,47] and in *Chryseobacterium* spp. [14,48]. While data are more limited for *Weeksella virosa*, it has been suggested that it may display intrinsic resistance to aminoglycosides as well [24].

While antimicrobial susceptibility testing data on *Bergeyella* spp. are limited due to the low number of recorded cases in the literature, broad susceptibility of isolates to β-lactams (including carbapenems), tetracyclines, and fluoroquinolones has been documented in case reports [34,35,37]. Notably, *Bergeyella zoohelcum* is known to display intrinsic resistance to colistin and polymixin B [49].

*Capnocytophaga* spp. can be broadly classified into human- or animal-associated species [50]. The antimicrobial susceptibility pattern of these varies greatly: for instance, there is some evidence that *C. canimorsus* displays regular penicillin sensitivity, while other animal-associated species show markedly divergent susceptibility profiles driven by β-lactamase expression [51]. Recent published data on the resistance profiles of *Capnocytophaga* spp. writ large are limited. A 1986 study of strains isolated from clinical samples showed broad susceptibility of isolates to clindamycin, amoxicillin-clavulanate, and imipenem; several cephalosporins and ciprofloxacin also generally showed good in vitro activity. The same isolates were notably uniformly resistant to aminoglycosides, polymixin B, and vancomycin [52]. More recent testing on clinical isolates raises concern for emerging resistance to metronidazole and ciprofloxacin [53] as well as increased rates of β-lactamase production [54].

High levels of carbapenem resistance have been noted in *Chryseobacterium* isolates, regardless of geographic region. Testing of fifty *Chryseobacterium* spp. isolates collected between 1997 and 2001 from the worldwide SENTRY program revealed only six that were susceptible to imipenem; the isolates were much more susceptible to newer fluoroquinolones, and in particular, only one isolate displayed resistance to levofloxacin in vitro [48]. Results from investigation of a more recent collection of 245 *Chryseobacterium* isolates showed frequent resistance to ceftazidime, cefepime, meropenem, and imipenem. However, greater than 90% of the same isolates were susceptible to trimethoprim–sulfamethoxazole and levofloxacin, and 97.8% of isolates were susceptible to minocycline [55].

Intrinsic resistance to carbapenems is also displayed among members of the genus *Elizabethkingia*. A collated analysis of *E. anophelis*, *E. miricola*, and *E. meningoseptica* clinical isolates from East Asia and North America showed that of 437 isolates, none displayed susceptibility to tested carbapenem antibiotics [45]. Investigation of clinical isolates has revealed that many harbor one or more chromosomal metallo-β-lactamases that are likely responsible for this finding [56]. Vancomycin is under investigation as a potential adjunctive therapeutic for infections caused by organisms within the genus *Elizabethkingia*, though concerns have been raised about high in vitro MICs [57] and high-quality clinical data are currently lacking.

There is a relative dearth of published literature examining susceptibility of pathogens within the order Flavobacteriales to more novel antimicrobial agents. Cefiderocol, ceftazidime-avibactam, and aztreonam-avibactam showed markedly poor in vitro activity against 22 clinical isolates of *Elizabethkingia* spp. from Taiwan [58]. However, separate published data from 197 *E. anophelis* clinical strains showed that both eravacycline and omadacycline had excellent in vitro activity as measured by broth microdilution [59].

### 3.3. Summary of Results

An abbreviated summary of the findings of this review is displayed in Table 1.

## 4. Discussion

Many of the current gaps in knowledge regarding the pathogenic organisms within Flavobacteriales are a result of the relative rarity of these pathogens; the lack of currently established antibiotic breakpoints for any species within Flavobacteriales is one such area. Available guidance currently lumps the interpretation of antimicrobial susceptibility data for pathogens in Flavobacteriales with other less commonly encountered Gram-negative pathogens, and it is not clear if this approach has significant clinical consequences. Further studies geared toward informing future official breakpoints for these organisms, including the outbreak-prone pathogens of the genus *Elizabethkingia*, are warranted.

Clinicians should be aware of the preponderance of carbapenem and aminoglycoside resistance among these organisms and tailor empiric therapy appropriately. Carbapenems and aminoglycosides should not be utilized as treatment for *Elizabethkingia*, *Myroides* or *Chryseobacterium* spp. infections, and deliberate and thoughtful selection of empiric antibiotic therapy for all pathogenic Flavobacteriales should be pursued. Further quality control initiatives and retrospective studies aimed at identifying and addressing the rate of inappropriate empiric antibiotic coverage of these organisms should be pursued.

With regard to efficacious treatments, fluroquinolones, trimethoprim–sulfamethoxazole, and minocycline generally appear to show promise as therapy for pathogenic Flavobacteriales that display resistance to β-lactams, including carbapenems. Minocycline appears to have robust in vitro activity against most clinical isolates of *Elizabethkingia* spp. and *Chryseobacterium* spp. Organisms of the genus *Capnocytophaga* appear to be more reliably susceptible to β-lactam antibiotics, though susceptibility profiles are evolving with time.

Even within a single species, the antimicrobial susceptibility profile of pathogens within Flavobacteriales appears to vary markedly by geographic region. This highlights the need for continued close global surveillance of the antibiotic resistance profiles of these pathogens. Additionally, available susceptibility data on pathogenic organisms within genera such as *Flavobacterium*, *Weeksella,* and *Empedobacter* are extremely limited, and researchers and clinicians would benefit from more comprehensive published data on these topics.

With relatively low numbers of patients with Flavobacteriales infections and the high frequency of comorbid conditions in affected patients, the categorization of predisposing conditions, the study of patient outcomes, and the examination of appropriate rates of empiric antibiotic coverage become quite challenging. The pathogens within Flavobacteriales share numerous commonalities, including a predisposition to cause disease in hospitalized and immunocompromised hosts; however, it should be noted that many of these pathogens have been reported to cause disease in immunocompetent hosts as well. The association of *Capnocytophaga* and *Bergeyella* spp. with the oral microbiome of animals and humans, as well as the association between *C. canimorsus* and sepsis in splenectomized patients, should be kept in mind by the clinician during investigation and history-taking. Further studies are also warranted to further clarify the relationship between the vaginal microbiome, female sex, and the incidence of infections caused by *Weeksella* spp.

While a full analysis of the clinical microbiology of Flavobacteriales is out of the scope of this review, it is worth noting that certain species have multiple phenotypic features in common with other organisms, which can potentially complicate identification. For instance, the slow-growing, nonmotile, oxidase-positive *Elizabethkingia anophelis* shares these features with organisms of the genus *Brucella*, which were only recently removed from the US government’s list of select agents [60]. The potential for such confusion to arise has significant implications for the safety of clinical laboratory staff and the speed of pathogen identification. Further complicating the identification of these organisms are the frequent changes in classification within the order. Some case reports have also remarked upon the failure of biochemical methods to identify organisms within Flavobacteriales, necessitating the use of modern methods such as 16S RNA sequencing for accurate identification; MALDI-TOF has shown variable success [19,27,28,37,49]. Clinical microbiology labs and clinicians would benefit from further investigation into the optimal diagnostic methods for identification of these pathogens.

Studies of newer antibiotic therapies on clinical isolates of *Elizabethkingia* spp. show poor bactericidal activity of cefiderocol and combination β-lactamase-avibactam therapies, though eravacycline and omadacycline show great promise. It remains to be elucidated whether cefiderocol or other Trojan-horse antibiotics [61] might play a role in the treatment of multidrug-resistant organisms in other pathogenic genera. Regarding these newer antibiotics, data from isolates outside of East Asia should be obtained, and testing of these drugs expanded to other pathogens within Flavobacteriales. Furthermore, given the extensive drug resistance displayed by many pathogens within the order, further investigation into molecular mechanisms of antibiotic resistance displayed by these pathogens is warranted, alongside exploration of possible alternative avenues of treatment such as bacteriophage therapy [62] or the targeting of bacterial metallophores [63].

## 5. Conclusions

There are multiple limitations of this narrative review. Included studies displayed marked heterogeneity in design; furthermore, included studies came from multiple geographic regions, potentially limiting the generalizability of results relating to clinical outcomes and antibiotic susceptibility data. The rarity of infections caused by Flavobacteriales means that available data in certain areas are quite limited, particularly in the case of the genera *Weeksella*, *Empedobacter*, *Flavobacterium*, and *Bergeyella*. Several older studies were also included due to the paucity of more recent published data on certain topics.

Organisms within the order Flavobacteriales represent rare but clinically important causes of infections in humans, and particularly in immunocompromised and hospitalized hosts. Resistance to multiple classes of antibiotics, and especially to aminoglycosides and carbapenems, among these organisms is common. Several species have also been implicated in serious outbreaks of illness. Due to their rarity, there are many gaps in current knowledge regarding human infections caused by Flavobacteriales, and future studies should further examine the clinical associations, antibiotic resistance patterns, and outcomes of these infections. Further laboratory investigations of the mechanisms of antibiotic resistance and virulence among clinical isolates of these organisms are also warranted.

## Figures and Tables

**Table 1:** Summary of Human Infections from Pathogenic Flavobacteriales.

Genus	Representative Species	Common Clinical Associations	Antibiotic Selection Considerations	References
*Elizabethkingia*	*E. anophelis*, *E. meningoseptica*, *E. miricola*	Bacteremia; meningitis	Intrinsic resistance to aminoglycosides and carbapenems; chromosomal metallo-β-lactamase production; vancomycin has been investigated as adjunctive therapy, although clinical evidence remains limited; eravacycline and omadacycline demonstrate excellent in vitro activity.	[7,8,9,10,11,12,13,40,44,45,56,57,58,59]
*Chryseobacterium*	*C. indologenes*, *C. gleum*	Bacteremia; pneumonia; infections associated with indwelling lines and devices	Frequent resistance to aminoglycosides, carbapenems, and cephalosporins; TMP-SMX, levofloxacin, and minocycline often demonstrate activity.	[7,14,15,38,48,55]
*Myroides*	*M. odoratus*, *M. odoratimimus*	Skin and soft tissue infection; osteomyelitis; urinary tract infection; bacteremia	Intrinsic resistance to aminoglycosides; multidrug resistance is common.	[7,16,17,18,19,39,46]
*Empedobacter*	*E. brevis*, *E. falsenii*	Bacteremia; endophthalmitis outbreak	Clinical data are limited; resistance to aminoglycosides and cephalosporins has been frequently reported.	[7,20,21,22,23]
*Weeksella*	*W. virosa*	Vaginal colonization; possible predilection for female patients	Possible intrinsic resistance to aminoglycosides.	[7,24,25]
*Flavobacterium*	*F. ceti*, *F. lindanitolerans*	Meningitis	Clinical experience is limited to isolated case reports.	[7,26,27,28,29,30,31]
*Bergeyella*	*B. zoohelcum*, *B. cardium*	Animal oral flora; bite wounds; endocarditis; possible human oral flora	*B. zoohelcum* is intrinsically resistant to colistin and polymyxin B; reported isolates are generally susceptible to carbapenems, tetracyclines, and fluoroquinolones, although clinical data remain sparse.	[7,33,34,35,36,37,49]
*Capnocytophaga*	*C. canimorsus*, *C. gingivalis*, *C. ochracea*, *C. sputigena*	Human and animal oral flora; bite wounds; endocarditis	Possible intrinsic resistance to aminoglycosides and polymyxin B; β-lactam susceptibility is variable; emerging resistance to metronidazole and ciprofloxacin has been reported.	[7,32,50,51,52,53,54]

## Data Availability

No new data were created or analyzed in this study. Data sharing is not applicable to this article.

## Abbreviations

AST	Antimicrobial Susceptibility Testing
CLSI	Clinical and Laboratory Standards Institute
EUCAST	European Committee on Antimicrobial Susceptibility Testing
MALDI-TOF	Matrix-Assisted Laser Desorption/Ionization Time-of-Flight
